# Nuclear and organelle genome assemblies of 5 *Cucumis melo* L. accessions, Ananas, Canton, PI 414723, Vedrantais, and Zhimali, belonging to diverse botanical groups

**DOI:** 10.1093/g3journal/jkaf098

**Published:** 2025-05-13

**Authors:** Javier Belinchon-Moreno, Aurelie Berard, Aurelie Canaguier, Isabelle Le-Clainche, Vincent Rittener-Ruff, Jacques Lagnel, Damien Hinsinger, Nathalie Boissot, Patricia Faivre-Rampant

**Affiliations:** Centre INRAE Île-de-France Versailles-Saclay, EPGV, Université Paris-Saclay, Evry F-91057, France; INRAE, Génétique et Amélioration des Fruits et Légumes, Montfavet 84143, France; Centre INRAE Île-de-France Versailles-Saclay, EPGV, Université Paris-Saclay, Evry F-91057, France; Centre INRAE Île-de-France Versailles-Saclay, EPGV, Université Paris-Saclay, Evry F-91057, France; Centre INRAE Île-de-France Versailles-Saclay, EPGV, Université Paris-Saclay, Evry F-91057, France; INRAE, Génétique et Amélioration des Fruits et Légumes, Montfavet 84143, France; INRAE, Génétique et Amélioration des Fruits et Légumes, Montfavet 84143, France; Centre INRAE Île-de-France Versailles-Saclay, EPGV, Université Paris-Saclay, Evry F-91057, France; INRAE, Génétique et Amélioration des Fruits et Légumes, Montfavet 84143, France; Centre INRAE Île-de-France Versailles-Saclay, EPGV, Université Paris-Saclay, Evry F-91057, France

**Keywords:** *Cucumis melo*, genome assembly, NLR, mitochondria, chloroplast, Ananas, Canton, PI 414723, Vedrantais, Zhimali

## Abstract

The construction of accurate whole genome sequences is pivotal for characterizing the genetic diversity of plant species, identifying genes controlling important traits, or understanding their evolutionary dynamics. Here, we generated the nuclear, mitochondrial, and chloroplast high-quality assemblies of 5 melon (*Cucumis melo* L.) accessions representing 5 botanical groups, using the Oxford Nanopore sequencing technology. The accessions here studied included varied origins, fruit shapes, sizes, and resistance traits, providing a holistic view of melon genomic diversity. The final chromosome-level genome assemblies ranged in size from 359 to 365 Mb, with approximately 25× coverage for 4 of them multiplexed in half of a PromethION flowcell, and 48× coverage for the fifth, sequenced individually in another half of a PromethION flowcell. Contigs N50 ranged from 7 to 15 Mb for all the assemblies, and very long contigs reaching sizes of 20–25 Mb, almost compatible with complete chromosomes, were assembled in all the accessions. Quality assessment through Benchmarking Universal Single-Copy Orthologs (BUSCO) and Merqury indicated the high completeness and accuracy of the assemblies, with BUSCO values exceeding 96% for all accessions, and Merqury QV values ranging between 41 and 47. We focused on the complex NLR resistance gene regions to validate the accuracy of the assemblies in highly complex and repetitive regions. Through Nanopore adaptive sampling, we generated accurately targeted assemblies of these regions with significantly higher coverage, enabling the comparison to our whole genome assemblies. Overall, these chromosome-level assembled genomes constitute a valuable resource for research focused on melon diversity, disease resistance, evolution, and breeding applications.

## Introduction

Cucurbits gather numerous species that are food crops, with melon (*Cucumis melo*) being notably important. Melon originated in Asia (Sebastian 2010) and a highly effective reproductive barrier isolated it from its relatives (Che and Adelberg 2020). Nevertheless, a diverse array of shapes, colors, and flavors is observed in melon fruits. Two melon subspecies have been described (*agrestis* and *melo*), and wild melons are observed in both (Pitrat 2013). However, this classification is strictly defined by the pubescence of the ovary (Kirkbride 1993) and is not always accurate (Pitrat 2013; Endl *et al*. 2018). A more precise classification is likely the botanical groups, which currently include 19 groups with several subgroups (Pitrat 2016). This classification is based on multiple phenotypic traits, and it generally correlates fairly well with geographic origins (Pitrat 2013, 2016). Many botanical groups include cultivated accessions grown in widely diverse regions (Pitrat 2016). A genomic representation of these groups would improve our understanding of adaptation to different growing conditions.

Like other cucurbits, melon faces many pests and diseases (Grumet *et al*. 2021). Plant breeders need therefore to screen a wide diversity of accessions to identify immunity loci that can be used in crop improvement programs. In plants, immunity traits are often encoded by resistance genes (R genes), commonly grouped in highly complex and diverse genome regions. Among R genes, nucleotide-binding site leucine-rich repeat resistance genes (NLRs) constitute the largest family (Barragan and Weigel 2021). In melon, the availability of reference genomes from different botanical groups would be highly beneficial for the identification of these genes.

Melon is a diploid species with a genome size under 400 Mb and organized into 12 chromosomes. To date, more than 40 melon genomes have been reported (Belinchon-Moreno *et al*. 2025), with different qualities and assembly levels, the last 3 being telomere-to-telomere (Li *et al*. 2023; Wei *et al*. 2023; Mo *et al*. 2024). The first assembled melon genome was released in 2012 (Garcia-Mas *et al*. 2012), and later refined using optical mapping (Ruggieri *et al*. 2018) and PacBio single-molecule-real-time (SMRT) sequencing technology (Castanera *et al*. 2020). Additional assemblies of diverse melon lines from different origins using long read sequencing (Zhang *et al*. 2019; Yano *et al*. 2020) suggested large genomic variations, notably on chromosomes 5, 6, and 10. A recent work by Oren *et al*. (2022) released the assembly of 24 accessions using ONT sequencing, and revealed differences between subspecies, including size disparities and large inversions, especially on chromosomes 1 and 11.

Technological improvements, particularly long-reads sequencing technologies, have increased the accuracy of genome assemblies (Rao *et al*. 2021). However, some complex regions like NLR clusters remain difficult to correctly assemble (Chovelon *et al*. 2021; Belinchon-Moreno *et al*. 2025). Targeted approaches, such as Nanopore adaptive sampling (NAS), hold promise for resolving putative assembly errors (Hook and Timp 2023; Belinchon-Moreno *et al*. 2025). These methods offer enhanced coverage in specific genome regions at a lower cost, facilitating more accurate genome assemblies of those areas.

Here, we report the nuclear, mitochondrial, and chloroplast genome assemblies of 5 diverse melon genomes, belonging to 5 botanical groups: 2 belonging to the subspecies *agrestis* and 3 to the subspecies *melo*. We evaluated the structural diversity among the 5 assemblies; we focused on the NLR gene regions to assess their accuracy in complex and repetitive genome areas. Finally, we compared our assemblies with the previously published version for 3 accessions, since random genetic changes over time and differences in gene bank conservation practices can result in significant genomic variation between accessions with the same name (Kameswara Rao *et al*. 2017).

## Materials and methods

### Sample selection

**Fig. 1.**
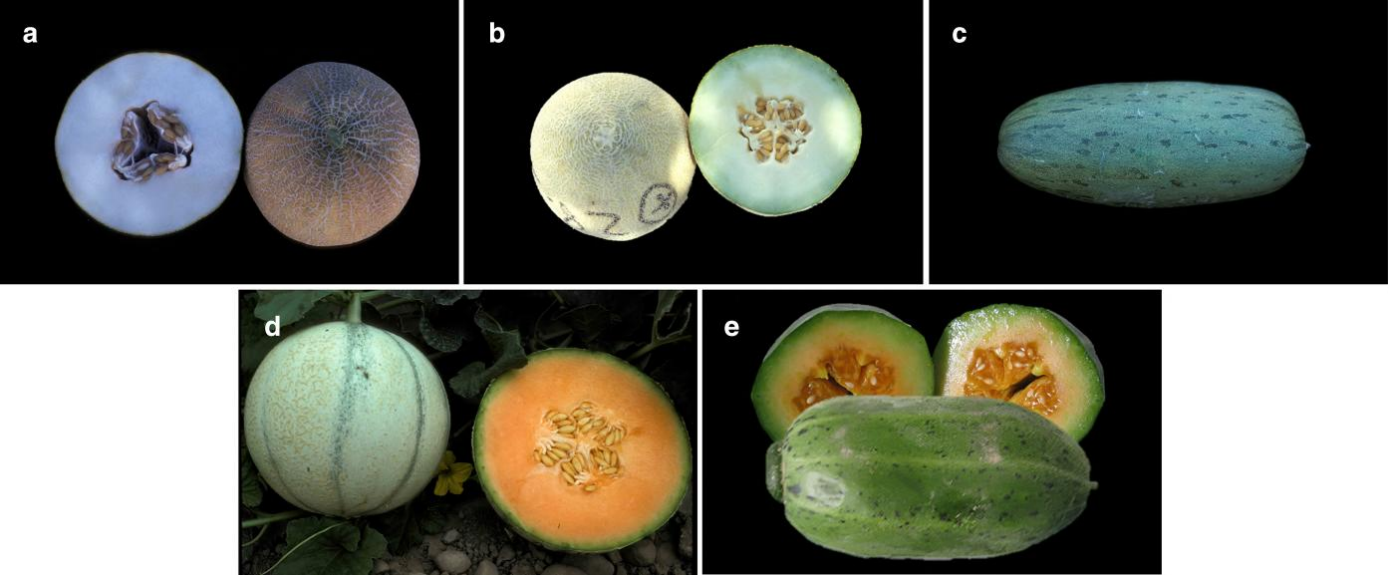
Mature fruits of the 5 selected accessions: a) Ananas (*ameri*); b) Canton (*reticulatus*); c) PI 414723 (*momordica*); d) Vedrantais (*cantalupensis*); and e) Zhimali (*chinensis*).

We selected 5 melon accessions from the subspecies *melo* (Ananas, Canton, and Vedrantais) and *agrestis* (PI 414723 and Zhimali). These accessions belong to 5 botanical groups: *ameri*, *reticulatus*, *cantalupensis*, *momordica*, and *chinensis*, respectively; and exhibit diversity of colors and shapes (Fig. 1). Their origin was located in Turkey, China (Guangdong), France, India (Uttar Pradesh), and China (Hebei), respectively. We recovered the seeds from the INRAE Center for Vegetable Germplasm in Avignon (Salinier *et al*. 2022) and cultivated in a greenhouse at INRAE GAFL, Avignon, France. The seeds were originally introduced to the INRAE Center for Vegetable Germplasm as follows: Canton in 2002 by ASL seeds company (Eyragues, France); Ananas in 1989 by Çukurova University (Adana, Turkey); PI 414723 in 1968 by USDA (La Jolla, California, USA); Vedrantais in 1982 by Vilmorin seeds company (La Ménitré, France); and Zhimali in 1994 by an unknown provider. We sampled young plant leaves and froze them in liquid nitrogen for subsequent DNA extraction. Genome assemblies of the accessions Ananas (Ananas Yoqne’am), Vedrantais, and PI 414723 were already published using ONT long reads for the 3 of them (Oren *et al*. 2022) and PacBio long reads for Ananas Yoqne’am (Vaughn *et al*. 2022).

### DNA Extraction, long-reads library preparation and sequencing

We extracted genomic DNA from 150 to 200 mg of young, fresh leaves previously frozen in liquid nitrogen. We used leaves from multiple plants, usually 4, depending on genotype leaf size. We used the NucleoSpin Plant II kit (Macherey-Nagel, Germany) following the manufacturer's protocol. We evaluated DNA quantity and quality using a Qubit4® 1x dsDNA BR Assay Kit (Invitrogen, Carlsbad, CA, USA) and an Agilent 2200 TapeStation (Agilent Technologies, Santa Clara, CA, USA). We carried out ONT libraries preparation and sequencing following ONT guidelines with the modifications described in Belinchon-Moreno *et al*. (2025). We used the Native Barcoding Kit 24 V14 (SQK-NBD114.24) (ONT, Oxford, UK). We sequenced the DNA of Canton using a whole PromethION *R10.4.1* flowcell (ONT, Oxford, UK). Moreover, we multiplexed and sequenced DNAs from Ananas, PI 414723, Vedrantais, and Zhimali using another PromethION *R10.4.1* flowcell (ONT, Oxford, UK). We used barcodes NB04, NB05, NB06, and NB07 for Zhimali, PI 414723, Vedrantais, and Ananas, respectively. In both experiments, we performed whole genome sequencing (WGS) using the channels 1,501–3,000 of the PromethION flowcells, keeping the channels 1–1,500 for a NAS approach. We used NAS to target 15 NLR regions detected with NLGenomeSweeper v. 1.2.1, following the process established in Belinchon-Moreno *et al*. (2025) and using the same reference genome, Anso77 (Supplementary Table 1). Briefly, we defined regions of interest (ROIs) by grouping predicted nucleotide-binding site (NBS) domains within 1 Mb and added 20 kb flanking buffers to ensure robust read depth. We excluded repetitive elements (REs) > 200 bp and interspersed sequences <500 bp located between 2 REs to optimize adaptive sampling. The final target regions without REs were used in MinKNOW for selective sequencing, accepting or rejecting reads based on initial ∼500 bp matches.

We extended both runs to 96 h. We performed a library reload (washing flush) in both experiments when the percentage of sequencing pores dropped to 20–30%. In the case of Canton, the library reload occurred 48 h after the start of the run. For the 4 multiplexed accessions, the reload was performed at 46 h. We set the sequencing speed to 260 bp (accuracy mode), and the quality score threshold to 10. We performed live base calling of the raw ONT FAST5 files with Guppy (ONT, London, UK) v. 6.3.9 for Canton and v. 6.4.6 for the 4 multiplexed samples in the “super accurate base-calling” mode. We used the MinKNOW software (ONT, London, UK) v. 22.10.7 for Canton and v. 22.12.5 for the 4 multiplexed DNAs. In this second run, we switched on the “trim barcodes” option to automatically trim the barcodes. For each run, we retained the “sequencing_summary.txt” file and the FASTQ files of the samples for further data processing.

### DNA Extraction, short-reads library preparation and sequencing

For PI 414723, Vedrantais, and Canton, Illumina (Illumina, San Diego, CA, USA) DNA libraries were prepared starting from DNA extracted with the Qiagen DNAeasy Plant miniKit (Qiagen, Valencia, CA, USA) and using the Kapa Hyper Prep Kit PCR-Free (KapaBiosystems, Wilmington, MA, USA), following the manufacturer's recommendations. The libraries were sequenced on an Illumina NovaSeq6000 instrument (Illumina, San Diego, CA, USA) using the 150 bp paired-end reads configuration.

For Ananas and Zhimali, MGI (MGI, Shenzhen, China) DNA libraries were prepared using the MGIEasy PCR-Free DNA Library Prep Kit (MGI, Shenzhen, China) and following the manufacturer's instructions. Briefly, 1 μg DNA was fragmented followed by a two-step size selection process using AMPure XP beads (Beckmann Coulter Genomics, Danvers, MA, USA). The quality and quantity of the fragmented and size-selected DNA were evaluated using an Agilent 2100 Bioanalyzer (Agilent Technologies, Santa Clara, CA, USA) and a Qubit4® 1x dsDNA HS Assay Kit (Invitrogen, Carlsbad, CA, USA), respectively. Then, the size-selected DNA was end-repared, 3′-adenlylated, and MGIEasy PF Adapters (MGI, Shenzhen, China) were ligated to it. The ligation products were purified using AMPure XP beads (Agilent Technologies, Santa Clara, CA, USA), and their quality and quantity were evaluated as previously described. Then, DNA was heat-denatured and the single-strand molecules were circularized. The remaining linear molecules were digested, obtaining a single-strand DNA library that was purified using AMPure XP beads (Agilent Technologies, Santa Clara, CA, USA) and quantified using a Qubit4® 1x ssDNA HS Assay Kit (Invitrogen, Carlsbad, CA, USA). Samples were pooled and DNA nano balls (DNBs) were produced and quantified with a Qubit4® 1x ssDNA HS Assay Kit (Invitrogen, Carlsbad, CA, USA). We loaded the DNBs into G400HM flowcells and sequenced them using an MGI DNBSEQ-G400RS device with a paired-end read length of 150 bp and the HotMPS High-throughput Sequencing chemistry.

### Heterozygosity estimation, data processing, and genome assembly

Prior to perform genome assemblies, we used the high-quality short-read sequencing data to calculate the 31-mer distribution with Jellyfish v. 2.3.1 (Marçais and Kingsford 2011). The k-mer histogram generated from the Jellyfish output was analyzed using GenomeScope2 (Ranallo-Benavidez *et al*. 2020) to estimate the heterozygosity rate of the genomes.

We performed data processing of ONT reads as described in Belinchon-Moreno *et al*. (2025). Briefly, we applied 2 filters to the raw ONT reads for each accession. First, we filtered out reads flagged as “FAIL”, meaning that their average quality score was lower than 10. Then, using the “sequencing summary” file, we selected those reads with a “Signal Positive” end reason, filtering out those labeled as “Data Service Unblock Mux Change” (reads rejected in NAS), “Unblock Mux Change”, “Mux Change”, and “Signal Negative”. For each accession, we split the reads by channel producing 2 separate FASTQ files: one with the reads sequenced on channels 1–1,500 for NAS, and another with the reads generated on channels 1,501–3,000 for WGS. We assessed statistics on the generated FASTQ files using Seqkit stats v. 2.4.0 (Shen *et al*. 2016).

For each accession, we assembled the filtered ONT reads longer than 10 kb from the WGS half-flowcell using the Flye assembler v. 2.9.1 (Kolmogorov *et al*. 2019). We used default parameters and added the options –nano-hq and –read-error 0.03, as suggested in the software guidelines for the last ONT Kit V14 chemistry. To correct potential sequencing errors, we polished the generated contigs using the short reads with Pilon v. 1.23 (Walker *et al*. 2014) with default parameters and adding the options –minmq 30 –minqual 30. We performed a reference-guided scaffolding of the generated contigs into pseudomolecules using RagTag v. 2.1.0 (Alonge *et al*. 2022) with the function “scaffold”. We used the genome assembly of Harukei-3 (Yano *et al*. 2020) as the reference for the nuclear and mitochondrial genomes. We filtered out the unanchored contigs shorter than 40 kb.

We assembled the plastome sequences separately using ptGAUL v. 1.0.5 (Zhou *et al*. 2023), with the GenBank accession MT622320 (Bai *et al*. 2021) as a reference to select chloroplast ONT reads. ptGAUL uses Flye to generate a chloroplast assembly graph and identifies distinct chloroplast paths within it. We filtered out unplaced contigs in the nuclear assembly matching the assembled chloroplast genome sequences.

Regarding NAS assemblies, filtered NAS reads longer than 5 kb were assembled using SMARTdenovo v. 2018.2.19 (Liu *et al*. 2021), using default parameters and adding the “generate consensus” option. We filtered generated contigs based on the presence of NBS domains as described in Belinchon-Moreno *et al*. (2025). We aligned the filtered NAS reads back to the NAS assemblies using Minimap2 v. 2.28 (Li 2018) with parameters “-ax map-ont -r2k” and manually inspected the alignments in IGV v. 2.17.1 (Robinson *et al*. 2011) to identify potential misassemblies or heterozygous variant calls. Using NLR annotation tools like NLRAnnotator v. 2.1b or RGAugury v. 2.2 (Li *et al*. 2016; Steuernagel *et al*. 2020), and analyzing the annotated NLR set in previously released *Cucumis melo* assemblies (Garcia-Mas *et al*. 2012; Zhang *et al*. 2019; Yano *et al*. 2020; Pichot *et al*. 2022; Mo *et al*. 2024), we discovered that 6 isolated NLR genes were missed in the reference used for adaptive sampling: 3 containing NBS domains, and 3 just containing TIR domains (Supplementary Table 1). To recover these genes, we used the rejected NAS reads along with the WGS short reads to perform reference-guided assemblies using the Anso77 genome assembly as a reference. We used the software Geneious Prime v. 2022.1.1 (http://www.geneious.com/) for this purpose. First, we obtained consensus sequences through the alignment of rejected NAS reads, and second, we polished the generated consensus assemblies using the WGS short reads.

We ran QUAST v. 5.0.2 (Gurevich *et al*. 2013) with default parameters to assess the metrics of the generated assemblies.

### Repeat annotation

We masked and annotated REs on the WGS assemblies using a hybrid strategy combining de novo prediction and homology-based annotation. First, we used RepeatModeler v. 2.0.2 (Flynn *et al*. 2020) to build a de novo repeat library using each genome assembly. We used default parameters and we added the option –LTRStruct to run the LTR structural discovery pipeline (LTR_Harvest and LTR_retriever) and combined the results with those obtained with the default RepeatModeler pipeline. Then, we performed homology-based predictions of the repeat library on the genome assembly using RepeatMasker v. 4.1.2 (Smit *et al*. 2015) with default parameters and adding the options -a –s and –gff. This pipeline allowed us to annotate and mask the genome assemblies.

### Gene prediction and functional annotation

We conducted gene prediction on the assembled nuclear genomes using 2 distinct methods. The first approach involved a combination of *ab initio*, homology-based, and transcriptome-based methods, integrated by EuGene v. 4.3 (Sallet *et al*. 2019). Annotation was performed using the eukaryote EGN-EP pipeline v. 2.0.3 (http://eugene.toulouse.inra.fr/), which encompasses various steps including probabilistic sequence model training, genome masking, computation of transcript and protein alignments, and detection of alternative splice sites. To aid in the identification of translated regions, we utilized 5 protein databases as evidence sources, including TAIR10, UniProtKB/Swiss-Prot, a plant subset of UniprotKB/TrEMBL, the proteome of *Cucumis melo* DHL92 v. 3.6.1, and a manually annotated subset of *Vat* proteins. Before alignment, sequences resembling those in REPBASE were excluded to ensure annotation precision. Transcriptome evidence included the meta-transcriptome used for Charmono melon genome annotation (Pichot *et al*. 2022), along with the coding sequence of manually annotated *Vat* genes (Chovelon *et al*. 2021). This pipeline also performs ribosomal DNA (rDNA) prediction using barrnap v. 0.9 (Seemann 2018) in accurate mode with infernal v. 1.1.4 (Nawrocki and Eddy 2013).

For the second approach, we employed an *ab initio* method utilizing deep-learning techniques, implemented with Helixer v. 0.3.3 (Stiehler *et al*. 2021; Holst *et al*. 2023). Annotations were restricted to the land plant lineage. The Helixer software was accessed at https://www.plabipd.de/helixer_main.html.

We searched for NLR loci using NLGenomeSweeper v. 1.2.1 (Toda *et al*. 2020), a tool that is able to predict NBS domains that might correspond to a complete or partial NLR gene. Based on Belinchon-Moreno *et al*. (2025) observations, we combined both annotations, keeping the genes annotated by Helixer in regions overlapping NBS domains, and taking the rest of the genes annotated by EuGene.

We used the EuGene approach to annotate mitochondrial assemblies. Concerning the chloroplast sequences, we annotated protein-coding, tRNA and rRNA genes using the web-based version of CHLOROBOX GeSeq (Tillich *et al*. 2017), available at https://chlorobox.mpimp-golm.mpg.de/geseq.html. We selected the options “Annotate plastid Inverted Repeat” and “Annotate plastid trans-spliced rps12”. We selected ARAGORN v. 1.2.38 (Laslett and Canback 2004) with default settings for tRNA annotation. We also drew circular chloroplast maps using OGDRAW (Greiner *et al*. 2019).

We performed a functional annotation of all the predicted genes using EggNOG-mapper v. 2.1.12 (Cantalapiedra *et al*. 2021) with default settings. This tool mapped the gene sequences against the EggNOG database v. 5.0 (Huerta-Cepas *et al*. 2019), which provides comprehensive functional and orthologous information.

### Quality assessment

We employed diverse tools and procedures to evaluate the quality and completeness of the genome assemblies. First, we identified telomeres and centromeres using the quarTeT software v. 1.1.6 (Lin *et al*. 2023). We used the quartet_teloexplorer.py script (Yunzhi *et al*. 2023) with default parameters and setting the plant clade to search for the plant telomere sequence TTTAGGG. To identify centromeres, we used the quartet_centrominer.py script (Yunzhi *et al*. 2023) with default settings and adding the repeat annotation and gene annotation files to improve performance.

Additionally, we determined the presence of the rDNA sequences in the ONT reads and in the assemblies by alignment of the assembled 45S rDNA sequence from the T2T Kuizilikjiz melon accession (Wei *et al*. 2023) and the 5S rDNA sequence from Waminal *et al*. (2014) (GenBank accession KF543344.1) using BLAST v. 2.15.0.

Then, we used Benchmarking Universal Single-Copy Orthologs (BUSCO) v. 5.2.2 (Simão *et al*. 2015) with the embryophyta_odb10 dataset, which consists of 1,614 BUSCOs. This allowed us to assess the presence of conserved single-copy orthologous genes within the assemblies and within the predicted gene models. Furthermore, we used Merqury v. 1.3 (Rhie *et al*. 2020) to estimate the consensus quality value (QV) and k-mer completeness of the assemblies using short reads. The QV serves as a log-scaled probability of error for the consensus base calls, providing insights into the accuracy of the assembled sequences.

Moreover, we used the assemblies obtained with NAS (with a higher read depth) to evaluate the accuracy of the whole genome assemblies in complex regions of the genome usually difficult to assemble, like NLR clusters. We focused on the well-studied *Vat* region (Chovelon *et al*. 2021) to evaluate this accuracy, and we performed manual annotations of the *Vat* regions following Chovelon *et al*. (2021) and Belinchon-Moreno *et al*. (2025).

Finally, we compared the genome structures of the assembled accessions by aligning each chromosome sequence of each accession to its relative chromosome sequence of the other assemblies. We generated dot plots between the accessions using the online version of D-Genies (Cabanettes and Klopp 2018) available at https://dgenies.toulouse.inra.fr/.

## Results and discussion

### DNA Sequencing and genome assembly

DNA extraction from ∼150 to 200 mg of young, fresh leaves yielded 6,160 ng for Canton, 8,673 ng for Zhimali, 10,994 ng for PI414723, 11,529 ng for Vedrantais, and 9,648 ng for Ananas. The DNA Integrity Number (DIN) was 7.5 for Canton and ranged from 9.1 to 9.5 for the other samples. Tapestation electropherograms showed a uniform size distribution without undesirable shouldering in all samples, with a maximum peak size consistently exceeding 53 kb. Given these DIN and fragment size distribution values, we considered the DNA quality to be sufficient for sequencing. ONT sequencing produced almost 57 Gb of raw Nanopore sequences from the half PromethION flowcell used for WGS of the 4 multiplexed samples, while nearly 50 Gb were obtained from the half-flowcell dedicated for WGS of Canton (Table 1). Concerning NAS sequencing, we obtained around 25 Gb in the half-flowcell of the 4 multiplexed samples, and around 29 Gb in the half-flowcell of Canton. We obtained a sequence depth of filtered ONT reads for the whole genome assemblies ranging from 24.14 to 27.72× for the 4 multiplexed samples. These reads presented an N50 size ranging from 24.08 to 26.88 kb. In the case of Canton, a sequence depth of 48.13× with a N50 of 18.94 kb was available for the whole genome assembly (Table 1). Regarding filtered reads for the NAS assemblies, we obtained a sequence depth on the target regions ranging from 138.92 to 182.41 for the 4 multiplexed samples, and a sequence depth of 199.66 for Canton. The N50 of these reads ranged from 21.11 to 23.69 kb for the 4 multiplexed samples, and was of 10.80 kb for Canton (Table 1). Many raw reads belonging to Canton were filtered out in both WGS and NAS approaches due to their poor quality or their very short size, producing a lower filtered data quantity than expected. This was probably due to the lower sequenced DNA quality (DIN of 7.5), or to technical factors such as a lower flowcell lot quality or an extra DNA fragmentation during library preparation. Supplementary Fig. 1 shows the higher sequence depth on the target regions in NAS compared with WGS for the 5 accessions, after applying a same read filter by size of 1 kb. These differences in sequence depth were in accordance with our previous results (Belinchon-Moreno *et al*. 2025), allowing a more precise assembly of these genomic areas.

**Table 1.** Metrics of long (WGS and NAS) and short reads for the 5 melon accessions.

	Accession	# of seq. (M)	Total size (Gb)	Cov. (X)	Average length (bp)	Max length (bp)	N50 (bp)	Q20 (%)	Q30 (%)
**Raw data WGS ONT**	Ananas	0.93	13	34.23	14,011	356,262	22,778	82.98	72.81
	Canton	12.03	49.78	131.00	4,139	756,444	9,495	76.85	66.98
	PI 414723	1	13.90	36.58	13,984	224,869	21,164	82.34	72.39
	Vedrantais	1.55	16.24	42.74	10,489	736,754	20,017	82.69	72.66
	Zhimali	0.98	13.85	36.44	14,162	399,640	23,292	83.89	73.74
**Filtered WGS ONT** **(>10 kb)**	Ananas	0.4	9.17	24.14	22,952	157,960	25,899	88.06	77.85
	Canton	1	18.29	48.13	18,155	185,983	18,938	88.11	77.69
	PI 414723	0.45	9.78	25.75	21,718	172,246	24,084	88.46	78.43
	Vedrantais	0.47	10.53	27.72	22,260	189,026	24,945	88.34	78.26
	Zhimali	0.41	9.73	25.61	23,511	209,302	26,881	88.30	78.18
**Raw data NAS ONT**	Ananas	6.82	5.34	—	782	161,614	596	83.56	73.12
	Canton	45.05	29.42	—	653	799,147	618	74.89	64.42
	PI 414723	7.35	5.81	—	790	139,679	599	82.73	72.53
	Vedrantais	11.39	8.14	—	715	197,565	573	83.76	73.38
	Zhimali	7.15	5.76	—	806	187,413	606	84.53	74.19
**Filtered NAS ONT** **(>5 kb)**	Ananas	97,931	1.61	139.90	16,449	130,303	23,539	88.40	78.28
	Canton	336,410	2.69	199.66	7,989	126,175	10,804	88.16	77.74
	PI 414723	111,302	1.77	152.09	15,869	139,679	21,708	88.82	78.87
	Vedrantais	148,065	2.02	182.41	13,664	152,813	21,110	88.71	78.72
	Zhimali	113,865	1.84	138.92	16,173	165,337	23,686	88.72	78.63
**Illumina/MGI data**	Ananas	71.09	10.64	28.00	150	150	150	95.94	90.26
	Canton	86.50	12.88	33.90	150	150	150	97.51	93.01
	PI 414723	115.97	17.29	45.50	150	150	150	97.60	93.30
	Vedrantais	43.56	6.43	16.92	150	150	150	97.91	94.22
	Zhimali	87.16	13.05	34.35	150	150	150	96.66	91.58

Prior to assembly, we used GenomeScope2 based on short reads to assess the heterozygosity level of the samples. K-mer spectrums showed a unimodal distribution with a single peak and no shoulder for all the accessions (Supplementary Fig. 2), indicating very low heterozygous states in the genomes. We assembled the filtered WGS ONT reads of each accession using Flye, with a subsequent polishing step with short reads using Pilon. We chose the Flye assembler due to its widely recognized superior performance with ONT sequences (Murigneux *et al*. 2020; Sun *et al*. 2021), although it produces collapsed assemblies of diploid genomes. Given the low heterozygosity reported for the accessions, we determined that a phased assembly would not offer significant additional information. We used BUSCO to test the importance of the short-reads polishing step by assessing the genome completeness of the initial contig assembly both before and after the polishing. BUSCOs were slightly better after polishing for Ananas, Canton, and PI 414723, keeping stable for Vedrantais and Zhimali (Supplementary Table 2). These findings suggested that the polishing step was not strictly necessary with the latest ONT Kit V14 technology, as previously reported in other studies (Sereika *et al*. 2022). Initial contig assemblies' sizes ranged from 359.35 to 369.76 Mb (Table 2). Canton presented the lowest number of contigs (149), while Ananas had the highest number (488). Looking at the N50 of the contigs, Ananas presented the lowest N50 (6.63 Mb), while Vedrantais showed the highest (15.12 Mb). We were able to produce some very long contigs for all accessions reaching sizes of 20–25 Mb, almost compatible with complete chromosomes. We performed the nuclear and mitochondrial anchoring of contigs using RagTag with the genome assembly of Harukei-3 as the reference. We made this choice based on the high quality of this assembly and to avoid possible assembly errors on chromosome 5 of Payzawat or 6 of DHL92, as previously established by Chovelon *et al*. (2021) and Oren *et al*. (2022). This allowed to anchor between 98.2% (for Ananas) and 99.6% (for Canton) of the total contigs size. We checked the suitability of Harukei-3 as the reference by scaffolding the *agrestis* accessions on the published *agrestis* genomes of HS (Yang *et al*. 2020) and PI 511890 (Mo *et al*. 2024) accessions. As expected, we saw no substantial differences between assemblies as our long contigs sizes allowed to span across structural variations that might exist between the references used for scaffolding (Supplementary Fig. 3). After filtering out unanchored contigs shorter than 40 kb or matching their chloroplast genome sequence, from 7 (for Canton) to 36 (for Ananas) unanchored contigs were preserved in the final assemblies. The final size of the nuclear assemblies ranged from 359.21 to 365.84 Mb, aligning with previous findings. Further detailed metrics of the different stages of the nuclear assemblies are summarized in Table 2. Our assemblies of Ananas and PI 414723 showed comparable metrics to previous published versions, while Vedrantais exhibited significant improvement. Compared to published assemblies of these accessions, our contig N50 was slightly lower for Ananas, slightly higher for PI 414723, and nearly twice as high for Vedrantais. In terms of contig count, we obtained nearly twice as many contigs as the best Ananas assembly, a similar number to the previous PI 414723 assembly, and almost 4 times fewer for Vedrantais.

**Table 2.** Quality metrics for various stages of the 5 melon whole genome assemblies.

		Ananas Yoqne’am (Vaughn *et al*. 2022)	Ananas Yoqne'am (Oren *et al*. 2022)	Ananas GAFL	PI 414723 (Oren *et al*. 2022)	PI 414723 GAFL	Vedrantais (Oren *et al*. 2022)	Vedrantais GAFL	Canton GAFL	Zhimali GAFL
	Subspecies	*melo*	*melo*	*melo*	*agrestis*	*agrestis*	*melo*	*melo*	*melo*	*agrestis*
	Botanical group	ameri	ameri	ameri	momordica	momordica	cantalupensis	cantalupensis	reticulatus	chinensis
**Initial contig assembly**	Assembly size contigs (Mb)	438.23	367.45	369.76	363.40	362.07	365.37	364.06	363.56	359.35
	# of contigs	2,301	280	488	230	291	528	163	149	171
	Largest contig (Mb)	—	22.86	23.43	22.82	23.24	22.55	26.82	23.34	19.48
	Contigs size N50 (Mb)	9.88	8.18	6.63	11.89	13.99	6.88	15.12	10.46	8.78
**Scaffolding**	Method	RagTag on DHL92 + MR1	RagTag on Harukei-3	RagTag on Harukei-3	RagTag on Harukei-3	RagTag on Harukei-3	RagTag on Harukei-3	RagTag on Harukei-3	RagTag on Harukei-3	RagTag on Harukei-3
	# of placed contigs	1,004*^a^*	118*^a^*	170	99*^a^*	136	163*^a^*	72	95	89
	Placed size (Mb)	379.89*^a^*	361.71*^a^*	363.11	357.47*^a^*	359.02	360.15*^a^*	361.84	362.17	357.16
	# of unplaced contigs	1,297*^a^*	162*^a^*	318	131*^a^*	155	365*^a^*	91	54	82
	Unplaced size (Mb)	58.36*^a^*	2.95*^a^*	6.65	3.61*^a^*	3.04	2.67*^a^*	2.22	1.39	2.19
	# of gap sequences	239	104	157	73	123	136	59	82	76
	Gap size (kb)	23.90	10.40	15.70	7.30	12.30	13.60	5.90	8.20	7.60
**Final genome**	Total assembly size (Mb)	438.25	367.46	365.84	363.40	360.31	365.38	363.86	363.18	359.21
	# of chromosomes	12	12	12	12	12	12	12	12	12
	N50 chromosomes (Mb)	30.92	29.91	29.82	30.36	29.04	29.80	29.97	29.73	29.07
	# of unplaced contigs after filter by size	1,297	162	36	131	15	365	18	7	18
	Unplaced size (Mb)	58.36	2.95	2.48	3.61	0.99	2.77	1.76	0.87	1.79
	Size of mitochondria (Mb)	—	2.72	2.66	1.99	2.70	2.41	2.11	2.28	1.92
	Size of chloroplast (kb)	—	82.06	155.78	148.29	156.28	55.29	155.79	155.98	155.99
	Merqury QV	43.88*^b^*	—	42.92	—	44.99	—	40.63	45.66	46.52
	K-mer completeness (%)	98.92*^b^*	—	96.34	—	98.45	—	99.01	98.59	98.56
	Complete and single-copy BUSCOs	1,574 (97.5%)	1,574 (97.5%)	1,574 (97.5%)	1575 (97.6%)	1,577 (97.7%)	1,573 (97.5%)	1,573 (97.5%)	1,573 (97.5%)	1,574 (97.5%)
	Complete and duplicated BUSCOs	18 (1.1%)	17 (1.1%)	17 (1.1%)	19 (1.2%)	17 (1.1%)	16 (1.0%)	17 (1.1%)	17 (1.1%)	16 (1.0%)
	Fragmented BUSCOs	11 (0.7%)	11 (0.7%)	11 (0.7%)	8 (0.5%)	8 (0.5%)	13 (0.8%) 12	12 (0.7%)	12 (0.7%)	11 (0.7%)
	Missing BUSCOs	11 (0.7%)	12 (0.7%)	12 (0.7%)	12 (0.7%)	12 (0.7%)	12 (0.7%)	12 (0.7%)	12 (0.7%)	13 (0.8%)
	Assembly completeness BUSCOs (% of 1,614 genes)	98.6	98.6	98.6	98.8	98.8	98.5	98.6	98.6	98.5
	# annotated genes	28,628	37,183	31,416	36,458	31,078	36,984	31,328	31,334	30,834

This table also includes the quality metrics for published assemblies that match the accession names of those assembled in this study.

^*a*^Placed and unplaced contigs and sizes after scaffolding were calculated from the provided FASTA files and assuming that no contig was filtered out.

^*b*^Results obtained from Mo *et al*. (2024).

GAFL, “*Génétique et Amélioration des Fruits et Légumes”* research unit, Avignon, France.

The size of the mitochondrial sequences ranged from 1.92 Mb for Zhimali to 2.7 Mb for PI 414723 (Table 2). The chloroplast sequence was independently assembled for each accession from selected ONT reads through ptGAUL. Two paths corresponding to the 2 orientations of the short single-copy (SSC) were obtained for Ananas, PI 414723, Vedrantais, and Zhimali, while only 1 path was assembled for Canton. The size of the chloroplast sequences (single path) ranged from 155.78 kb for Ananas to 156.28 kb for PI 414723 (Table 2). These sizes were similar to those found in the assembly by Wei *et al*. (2023).

We performed the assembly of the filtered ONT NAS reads using SMARTdenovo. As reported with other melon accessions (Belinchon-Moreno *et al*. 2025), we obtained very contiguous assemblies of the target regions, each represented by a single contig in the final assemblies. Manual inspection of the reads mapped back to the NAS assemblies showed very few polymorphisms, further confirming the accuracy of the NAS assemblies and the low heterozygosity of the samples. Detailed metrics of the NAS assemblies are summarized in Supplementary Table 3.

### Quality assessment

We used multiple strategies to evaluate the quality and completeness of the assembled genomes. First, we analyzed the assembly accuracy of tandem repetitive regions in the assemblies. These regions, such as centromeres, telomeres, and rDNA loci, are often challenging to assemble, leading to gaps in genome sequences (Wei *et al*. 2023). We predicted centromeric and telomeric sequences by searching tandem repeats and motifs in the genome assemblies. We identified the start and end positions of all 12 centromeres across all the assemblies (Supplementary Table 4). Additionally, we observed 22 out of 24 telomeres for Ananas, Canton, PI 414723, and Vedrantais, and 23 for Zhimali (Supplementary Table 4). All the assemblies presented successfully assembled telomeres at both ends in at least 10 out of the 12 chromosomes. These results indicated a higher level of completeness compared to the reference used for scaffolding (Harukei-3), where we identified a total of 14 telomeres, with 5 chromosomes presenting both of them (Supplementary Table 4).

We assessed the completeness of rDNA sequences in the assemblies by aligning the 45S and 5S rDNA sequences from Wei *et al*. (2023) and Waminal *et al*. (2014) to the ONT reads and to the assemblies. After 45S rDNA sequence alignment, we found 12 (for Canton) to 32 (for Ananas) hits with >90% identity and >5,000 bp alignment (Supplementary Table 5). The number of hits on the ONT reads corrected by the coverage ranged from 22.2 (for Canton) to 59.9 (for Ananas), indicating that around half of 45S rDNA gene copies were represented in the assemblies. As reported in the assembly of Wei *et al*. (2023), most 45S rDNAs were located on clusters on chromosomes 4 and 10, although in Zhimali and Vedrantais over half of them were located on contigs not assigned to chromosomal location (Supplementary Table 6). Moreover, we found 95 (for Vedrantais) to 222 (for Canton) hits with >90% identity and >100 bp match after 5S rDNA alignment to the assemblies (Supplementary Table 5). Similarly, around 50% of the rDNA loci identified in the reads were also present in the assemblies, with the exception of PI 414723, where we found more hits in the assembly than in the ONT reads after correcting for sequence depth (Supplementary Table 5). All hits across the assemblies aligned to chromosome 12, forming a 5S rDNA array with sizes ranging from 30.1 in Vedrantais to 108.6 kb in PI 414723. This 5S rDNA array was likewise reported in the genome assembly by Wei *et al*. (2023), but with an extended length of 1.8 Mb.

We subsequently searched for the conserved gene orthologs within the assemblies. We used the embryophyta_odb10 database for comparison purposes, as this database has been employed in the most recently published melon genome reports (Vaughn *et al*. 2022; Li *et al*. 2023; Wei *et al*. 2023; Mo *et al*. 2024). From the 1,614 conserved single-copy genes in the embryophyta_odb10 database, complete BUSCO scores ranged from 98.5 (for Zhimali) to 98.8% (for PI 414723) in the 5 assemblies. 0.5–0.7% orthologous genes were assigned as fragmented, and 0.7–0.8% were assigned as missing (Table 2). These results of complete BUSCOs were similar to those observed in previously assembled melon genomes using long reads (Wei *et al*. 2023), including the accessions with the same name published by Oren *et al*. (2022) and Vaughn *et al*. (2022) (Table 2).

As short paired-end reads were available for the 5 assembled genomes, we performed a second assessment of quality and completeness using Merqury. The consensus QV ranged from 40.63 (for Vedrantais) to 46.52 (for Zhimali), meaning that the accuracy was superior to 99.99% for all the assemblies. The k-mer completeness ranged from 96.34 (for Ananas) to 99.01% (for Vedrantais), respectively. These results were comparable to the best-assembled melon genomes published to date (Mo *et al*. 2024).

**Fig. 2.**
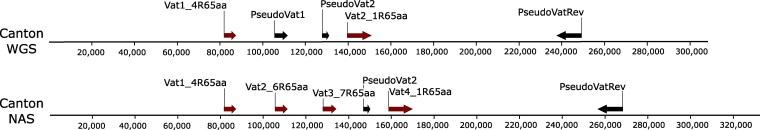
Manual annotation of the well-studied *Vat* region of Canton in the WGS (top) and NAS (bottom) assemblies.

A last assessment of assembly quality was performed focusing on the identification of NLR resistance genes in the complete genomes and in the NAS assemblies. The NAS assemblies were generated with significantly higher coverage (Supplementary Fig. 1), ensuring high accuracy, which we confirmed by a read alignment to the assemblies revealing very few polymorphisms. We searched for the conserved NBS domains using the software NLGenomeSweeper. Fifteen regions consisting of 9–10 clusters of NBS domains and 5–6 isolated NBS domains were identified in all the assemblies, in the same chromosome regions (Supplementary Table 7a–e). These results aligned with those previously obtained in other melon accessions (Belinchon-Moreno *et al*. 2025). For Ananas, PI 414723, Vedrantais, and Zhimali, the same number of NBS domains and in the same order were identified in the NAS and WGS assemblies (Supplementary Table 7a, c, d, and e), confirming the accuracy of these last. For Canton, 2 extra NBS domains were predicted in the NAS assembly: one in Region08 (where *Vat* is located) and another in Region13 (Supplementary Table 7b). Further analysis of the well-studied *Vat* region between markers M5 and M4 (Chovelon *et al*. 2021) by manual annotation of the *Vat* genes showed one extra *Vat* gene in the NAS assembly of Canton (Fig. 2). An assembly error in the WGS set probably caused the combination of *Vat2* and *Vat3* genes into a pseudogene (*PseudoVat1*). Long reads overpassing the concerned area, as well as the translation of *Vat2* and *Vat3* genes into 2 functional *Vat* proteins, demonstrated the veracity of the NAS assembly. These results confirmed the high accuracy of our WGS assemblies in highly complex areas such as NLR regions, as nearly all predicted NBS domains were consistent between both NAS and WGS assemblies across all the accessions. In addition, the results of Canton highlighted the benefits of using NAS in complex genome regions, since its higher sequence depth output allowed to produce a better assembly, even though the WGS assembly was built with a sequence depth of 48.13×.

### Repetitive content and gene annotation

The total number of annotated repetitive sequences using RepeatModeler varied from 658,089 (for PI 414723) to 674,522 (for Ananas). They represented a total size ranging from 206.42 (for PI 414723) to 211.83 Mb (for Ananas). In all the accessions, the total size of annotated repetitive sequences represented between 57 and 59% of the complete genome assembly size. Between 6,981 (in Zhimali) and 10,024 (in PI 414723) REs were annotated in the mitochondrial sequence, representing around 35% of the sequence length. In the chloroplast, the number of annotated REs ranged from 83 (in Zhimali) to 143 (in Vedrantais), representing around 80% of the sequence length. We compiled detailed information on the various categories of annotated REs within the nuclear, mitochondrial, and chloroplast sequences in Supplementary Table 8.

We annotated protein-coding genes using a combination of EuGene and Helixer. The total number of annotated genes ranged from 30,961 for Zhimali to 31,543 for Ananas. These numbers were consistent with previously published melon genome annotations (Garcia-Mas *et al*. 2012; Zhang *et al*. 2019; Yano *et al*. 2020; Pichot *et al*. 2022). From 30,638 to 31,198 gene features were annotated in the nuclear genomes. Of these, 92.16 (for PI 414723) to 92.74% (for Ananas) were functionally annotated with EggNOG. The average length of gene models in the nuclear genomes ranged from 3,498 (for PI 414723) to 3,553 bp (for Vedrantais), a bit longer than the previous results obtained in Payzawat (3,166 bp) and DHL92 v. 3.6.1 (2,326 bp). The average number of exons per gene in the nuclear genomes was around 4.7 for all the accessions. In total, more than 99% of the predicted protein-coding genes were allocated to the 12 chromosomes in all the assemblies. Complete BUSCO scores for the annotated protein-coding gene models were consistently around 95.5% across all accessions. 2.97–3.10% BUSCO orthologous genes were assigned as fragmented, and 1.43–1.67% were missing. These results were comparable to those obtained in other melon genomes assembled using long reads (Yano *et al*. 2020; Pichot *et al*. 2022; Li *et al*. 2023; Wei *et al*. 2023).

We predicted 69 (in Zhimali) to 96 (in PI 414723) genes in the mitochondrial sequences. Of them, around 50% were functionally annotated in all the assemblies using EggNOG. We annotated the chloroplast genomes independently using the specialized tool CHLOROBOX GeSeq. The number of annotated genes was 127 or 128 in all the assemblies considering a single path corresponding to one orientation of the inverted repeat (IR) regions. All chloroplast assemblies exhibited the typical tetrapartite organization, with a large single-copy (LSC), a small single-copy (SSC), and 2 IR regions (Supplementary Figs. 4 and 5). Supplementary Table 9 summarizes the annotation metrics of the 5 genome assemblies.

### Comparative analysis

The reported low heterozygosity rate of the accessions (Supplementary Fig. 2) ensured that Flye consensus assemblies contained all structural variations without loss or unintended merging. Additionally, the assembled long primary contigs (Table 2) already captured major structural variations, meaning that RagTag scaffolding primarily served to order contigs into linkage groups. Confident in the accuracy and completeness of our assemblies, we conducted a detailed structural comparison to further investigate genomic variation. We performed chromosome-to-chromosome alignments among the 5 genome assemblies using D-Genies. In general, we found more co-linearity when comparing genomes from the same subspecies (*agrestis* and *melo*). Nevertheless, structural variants specific to each accession appeared in all comparisons. Large intra-chromosomal inversions located in chromosomes 1 and 11 and spanning across 1.6 and 3.2 Mb appeared when comparing the assembly of Zhimali with those of all accessions belonging to the *melo* subspecies (Supplementary Figs. 6 and 7). This is a further evidence in support of the results presented by Oren *et al*. (2022), where they pointed out these 2 inversions as variants between most *agrestis* and *melo* accessions. Furthermore, as reported in the study of Oren *et al*. (2022), these 2 inversions were not found in the genome of PI 414723 (*agrestis* accession), confirming their findings. We also found an inversion spanning across 1.6 Mb in chromosome 7 that differentiated our assembled *melo* and *agrestis* genomes (Supplementary Fig. 8). However, we did not consistently find it when examining other previously published melon assemblies.

**Fig. 3.**
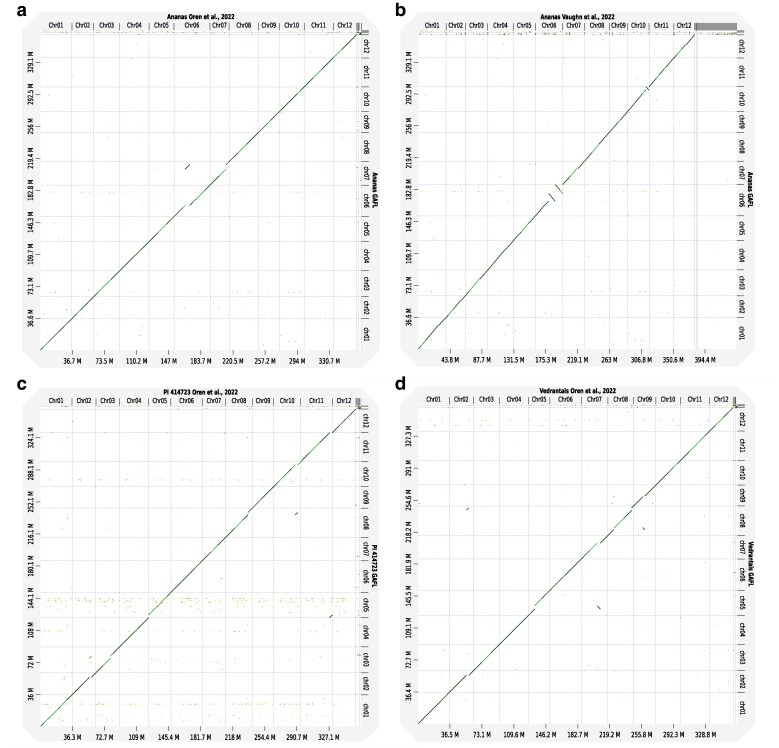
Chromosome-to-chromosome alignments between the whole genome assemblies constructed here (*y*-axis) and those previously published by Oren *et al*. (2022) or Vaughn *et al*. (2022) (*x*-axis). a) Ananas GAFL vs Ananas Oren *et al*. (2022). b) Ananas GAFL vs Ananas Vaughn *et al*. (2022). c) PI 414723 GAFL vs PI 414723 Oren *et al*. (2022). c) Vedrantais GAFL vs Vedrantais Oren *et al*. (2022). GAFL, “*Génétique et Amélioration des Fruits et Légumes*” research unit, Avignon, France.

We also compared the assemblies of Ananas, PI 414723, and Vedrantais to their previously published counterparts, noting some differences. For Ananas, a 5.2 Mb fragment on chromosome 6 of the published assembly by Oren *et al*. (2022) was placed on chromosome 7 in ours (Fig. 3a). This fragment in our assembly corresponded to an isolated contig, suggesting difficult-to-assemble regions at both ends. Comparison to the assembly of Vaughn *et al*. (2022) revealed 2 large inversions on chromosome 6, and another on chromosome 10 (Fig. 3b). The inversions on chromosome 6 likely resulted from the use of the DHL92 v. 3.6.1 genome as a reference for scaffolding, combined with shorter contigs that were unable to span these structural variations. Regarding PI 414723, significant structural variations were also identified between the 2 assemblies (Fig. 3c). A 2.4 Mb fragment from chromosome 2 in the published assembly was placed on chromosome 3 in ours. Additionally, a 3.1 Mb fragment at the end of chromosome 11 in the previous version was found at the beginning of chromosome 5 in our assembly. A 2.7 Mb fragment originally on chromosome 10 was relocated to chromosome 8, and a 1.2 Mb inversion was noted on chromosome 4. In the Vedrantais assemblies, 3 large translocations were observed (Fig. 3d). A 3 Mb fragment on chromosome 2 of the previous assembly was placed on chromosome 8 in ours. Similarly, a 3.7 Mb fragment on chromosome 7 in the earlier version was found on chromosome 5 in our assembly. Additionally, a 2 Mb fragment originally on chromosome 9 was relocated to chromosome 8. For all these genomic rearrangements, the assemblies generated in this study were congruent with those of Harukei-3, Payzawat, DHL92, and Charmono, suggesting their accuracy.

Focusing on NLR regions, the NLR region-to-region alignment of Ananas and Vedrantais to those from the accessions with the same name presented by Oren *et al*. (2022) and Vaughn *et al*. (2022) showed high similarity (Supplementary Fig. 9a, b, and d). However, significant structural variations in chromosome 1 and sequence divergence in chromosome 5 were observed between the NLR regions of both PI 414723 genome versions (Supplementary Fig. 9c). Manual annotation of the M5-M4 *Vat* zone from our assemblies and those of Oren *et al*. (2022) and Vaughn *et al*. (2022) showed identical gene structure and order for Ananas and PI 414723 accessions (Supplementary Fig. 10). However, one extra gene *Vat* (*Vat3*_2R65aa) was found in our assembly of Vedrantais (Supplementary Fig. 10). It corresponded to an insertion of 20 kb not found in the public assembly. All reported differences from published assemblies with the same name may result from assembly errors or, more likely, from genetic divergence following their introduction into different gene banks with different conservation practices. Regeneration of seed accessions is crucial for germplasm collection management, but it involves the risk of losing the genetic integrity of accessions due to genetic shift produced by unintended out-crossing and genetic drift from different multiplication practices or handling errors (Kameswara Rao *et al*. 2017). Having a genome assembly that precisely matches the genomic sequence of our seeds allows to understand this divergence, and it is essential for ensuring accuracy in downstream analyses, such as genetic association studies.

## Supplementary Material

jkaf098_Supplementary_Data

## Data Availability

The sequencing data (ONT and short reads) and final consensus sequences of the nuclear, mitochondrial, and chloroplast genomes of the 5 accessions are available at the NCBI database under the following BioProjects: PRJNA1164660 (Ananas), PRJNA1164662 (Canton), PRJNA1164667 (PI 414723), PRJNA1164698 (Vedrantais), PRJNA1164664 (Zhimali). Ananas: BioSample SAMN43903213, Accessions SRR30814798 and SRR30814797 for sequencing reads, and JBIEKO000000000 for genome assembly. Canton: BioSample SAMN43903216, Accessions SRR30815175 and SRR30815174 for sequencing reads, and JBHZJR000000000 for genome assembly. PI 414723: BioSample SAMN43903214, Accessions SRR30814801 and SRR30814800 for sequencing reads, and JBHZJT000000000 for genome assembly. Vedrantais: BioSample SAMN43903215, Accessions SRR30814803 and SRR30814802 for sequencing reads, and JBHZJU000000000 for genome assembly. Zhimali: BioSample SAMN43903212, Accessions SRR30806113 and SRR30806112 for sequencing reads, and JBHZJS000000000 for genome assembly. The structural and functional gene annotations of the 5 whole genome assemblies, as well as the repeat elements annotations are available at the Recherche Data Gouv database (https://entrepot.recherche.data.gouv.fr/) under DOI https://doi.org/10.57745/JPL1RC (Belinchon-Moreno *et al*. 2024a). The NAS targeted sequencing data are available in the NCBI database under BioProject PRJNA1127998. Sequencing reads accessions are SRR30783378 (Ananas); SRR30783375 a (Canton); SRR30783377 (PI 414723); SRR30783376 (Vedrantais); and SRR30783379 (Zhimali). The BioSamples are the same as those specified for the WGS data. The targeted assemblies and structural gene annotations of targeted regions are available at the Recherche Data Gouv database under DOI https://doi.org/10.57745/ZALVPU (Belinchon-Moreno *et al*. 2024b). The large sequencing_summary.txt files used for read filtering by end reason prior assembly are available from the corresponding author on reasonable request. The scripts used for data processing, assembly, and annotation are available in the GitHub repository indicated in the script_availability.txt file at the Recherche Data Gouv project with DOI https://doi.org/10.57745/JPL1RC (Belinchon-Moreno *et al*. 2024a). Supplemental material available at G3 online.
