# Supplementary material for: Nuclear and organelle genome assemblies of 5 Cucumis melo L. accessions, Ananas, Canton, PI 414723, Vedrantais, and Zhimali, belonging to diverse botanical groups
Source: G3 (Bethesda). 2025 May 13;15(7):jkaf098. doi: 10.1093/g3journal/jkaf098 (PMC12239611; doi:10.1093/g3journal/jkaf098)
Supplement: jkaf098_Supplementary_Data [file jkaf098_supplementary_data.zip › Figure_S10_G3-2025-405864.docx]

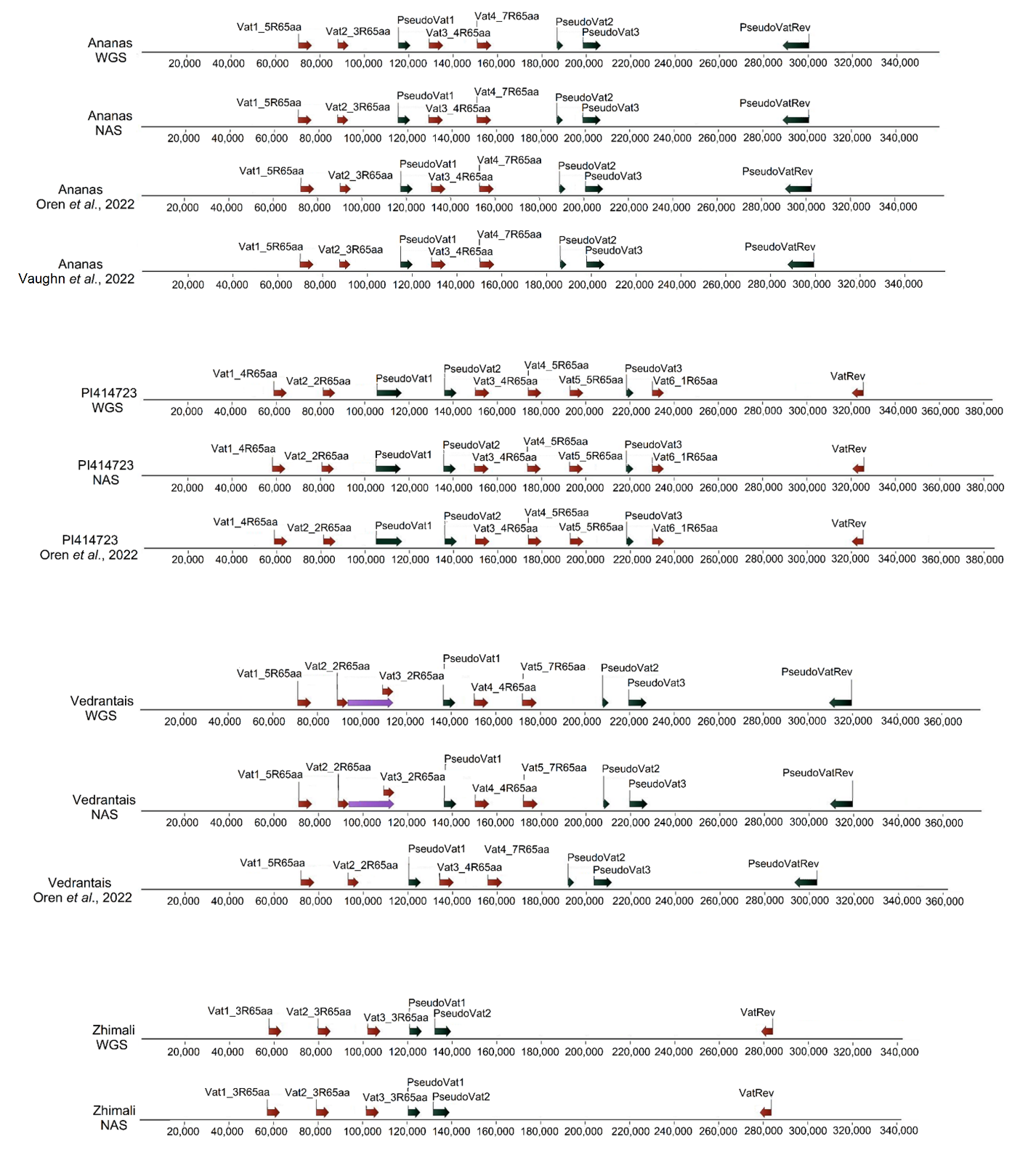


**Figure S10.** Manual annotation of the well-studied *Vat* region of Ananas, PI 414723, Vedrantais and Zhimali. We show the manual annotation of the WGS and NAS assemblies constructed here, as well as those previously published by Oren et al. (2022) and Vaughn et al. (2022) when they were available. For Vedrantais, the purple arrows represent an insertion found in both WGS and NAS assemblies compared to the previously published assembly.
