# Supplementary material for: Nuclear and organelle genome assemblies of 5 Cucumis melo L. accessions, Ananas, Canton, PI 414723, Vedrantais, and Zhimali, belonging to diverse botanical groups
Source: G3 (Bethesda). 2025 May 13;15(7):jkaf098. doi: 10.1093/g3journal/jkaf098 (PMC12239611; doi:10.1093/g3journal/jkaf098)
Supplement: jkaf098_Supplementary_Data [file jkaf098_supplementary_data.zip › Figure_S3_G3-2025-405864.docx]

**
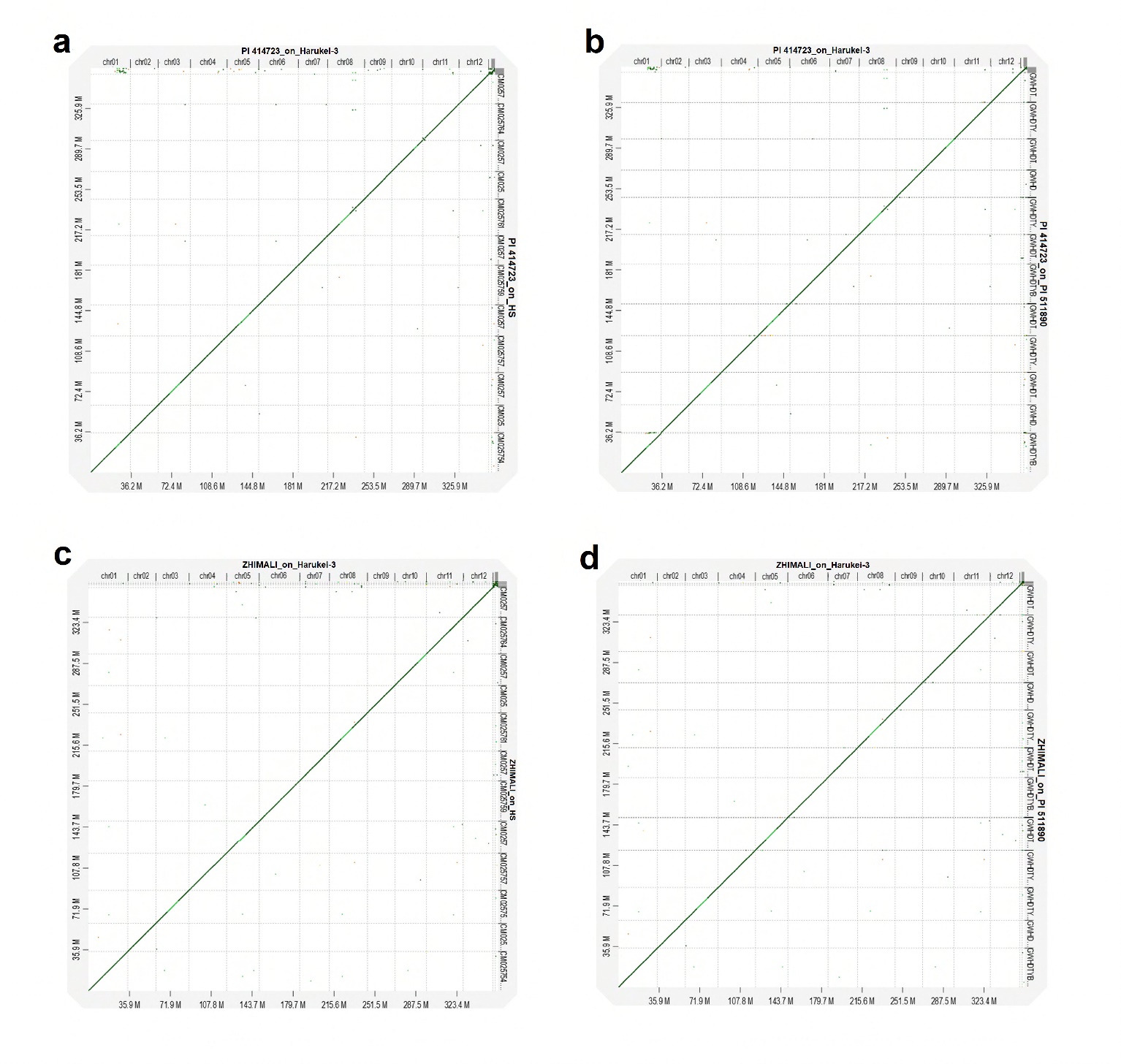
Figure S3.** Dot plots comparing the scaffolding of *agrestis* accessions on Harukei-3, and on two published *agrestis* genome assemblies: HS and PI 511890. A) PI 414723 scaffolded on Harukei-3 (x-axis) and HS (y-axis). B) PI 414723 scaffolded on Harukei-3 (x-axis) and PI 511890 (y-axis). C) Zhimali scaffolded on Harukei-3 (x-axis) and HS (y-axis). D) Zhimali scaffolded on Harukei-3 (x-axis) and PI 511890 (y-axis).
