# Supplementary material for: Nuclear and organelle genome assemblies of 5 Cucumis melo L. accessions, Ananas, Canton, PI 414723, Vedrantais, and Zhimali, belonging to diverse botanical groups
Source: G3 (Bethesda). 2025 May 13;15(7):jkaf098. doi: 10.1093/g3journal/jkaf098 (PMC12239611; doi:10.1093/g3journal/jkaf098)
Supplement: jkaf098_Supplementary_Data [file jkaf098_supplementary_data.zip › Figure_S4_G3-2025-405864.docx]

**
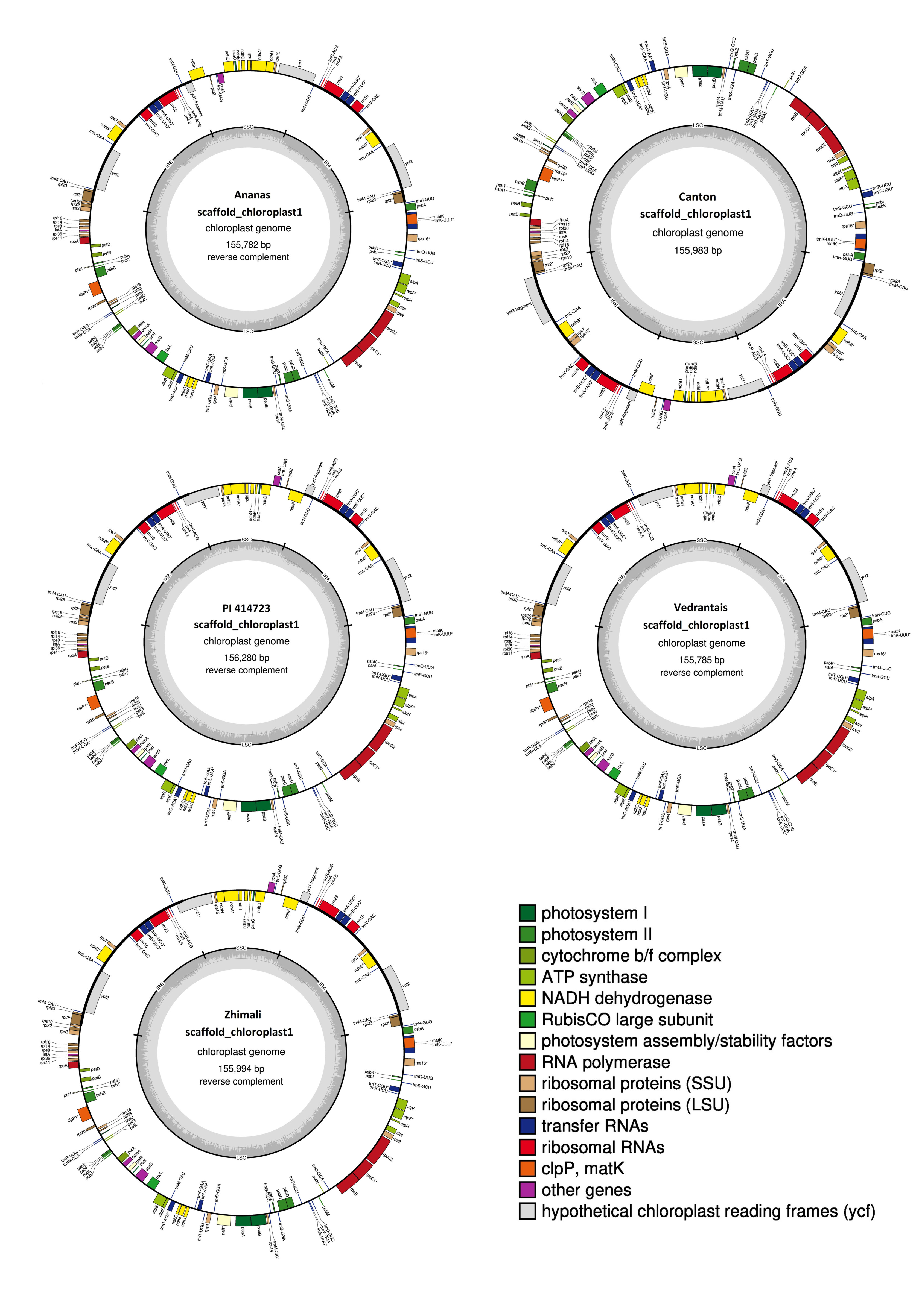
**

**Figure S4.** Circular diagrams depicting the first assembly path of the five chloroplast genomes, corresponding to one orientation of the SSC region. The diagram highlights the quadripartite structure, labeling the LSC, SSC, IRA, and IRB regions based on their defined boundaries. Within the inner circle, the light grey area indicates the AT content, while the darker grey layer represents the GC content. Genes situated inside the circle are transcribed in a clockwise direction, whereas those outside are transcribed counterclockwise. The genes are color-coded according to their functional groups. Genes with an asterisk denote genes containing introns.
