# Supplementary material for: Nuclear and organelle genome assemblies of 5 Cucumis melo L. accessions, Ananas, Canton, PI 414723, Vedrantais, and Zhimali, belonging to diverse botanical groups
Source: G3 (Bethesda). 2025 May 13;15(7):jkaf098. doi: 10.1093/g3journal/jkaf098 (PMC12239611; doi:10.1093/g3journal/jkaf098)
Supplement: jkaf098_Supplementary_Data [file jkaf098_supplementary_data.zip › Figure_S6_G3-2025-405864.docx]

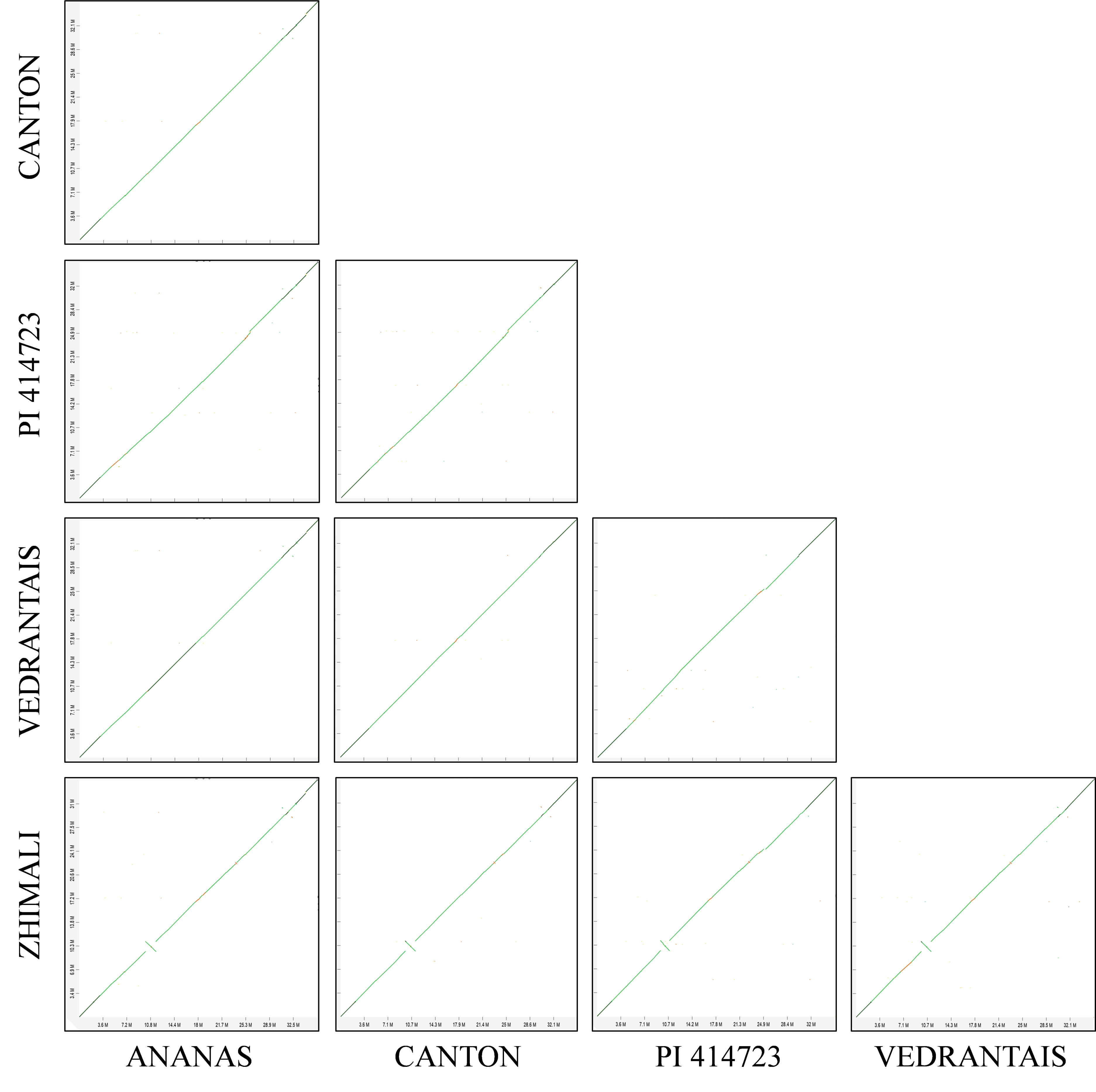


**Figure S6.** Chromosome-to-chromosome alignments between the chromosome 1 of the five genome assemblies. A large intra-chromosomal inversions spanning across 1.6 Mb appeared when comparing the assembly of Zhimali with the rest of accessions.
