# Supplementary material for: Nuclear and organelle genome assemblies of 5 Cucumis melo L. accessions, Ananas, Canton, PI 414723, Vedrantais, and Zhimali, belonging to diverse botanical groups
Source: G3 (Bethesda). 2025 May 13;15(7):jkaf098. doi: 10.1093/g3journal/jkaf098 (PMC12239611; doi:10.1093/g3journal/jkaf098)
Supplement: jkaf098_Supplementary_Data [file jkaf098_supplementary_data.zip › Figure_S9_G3-2025-405864.docx]

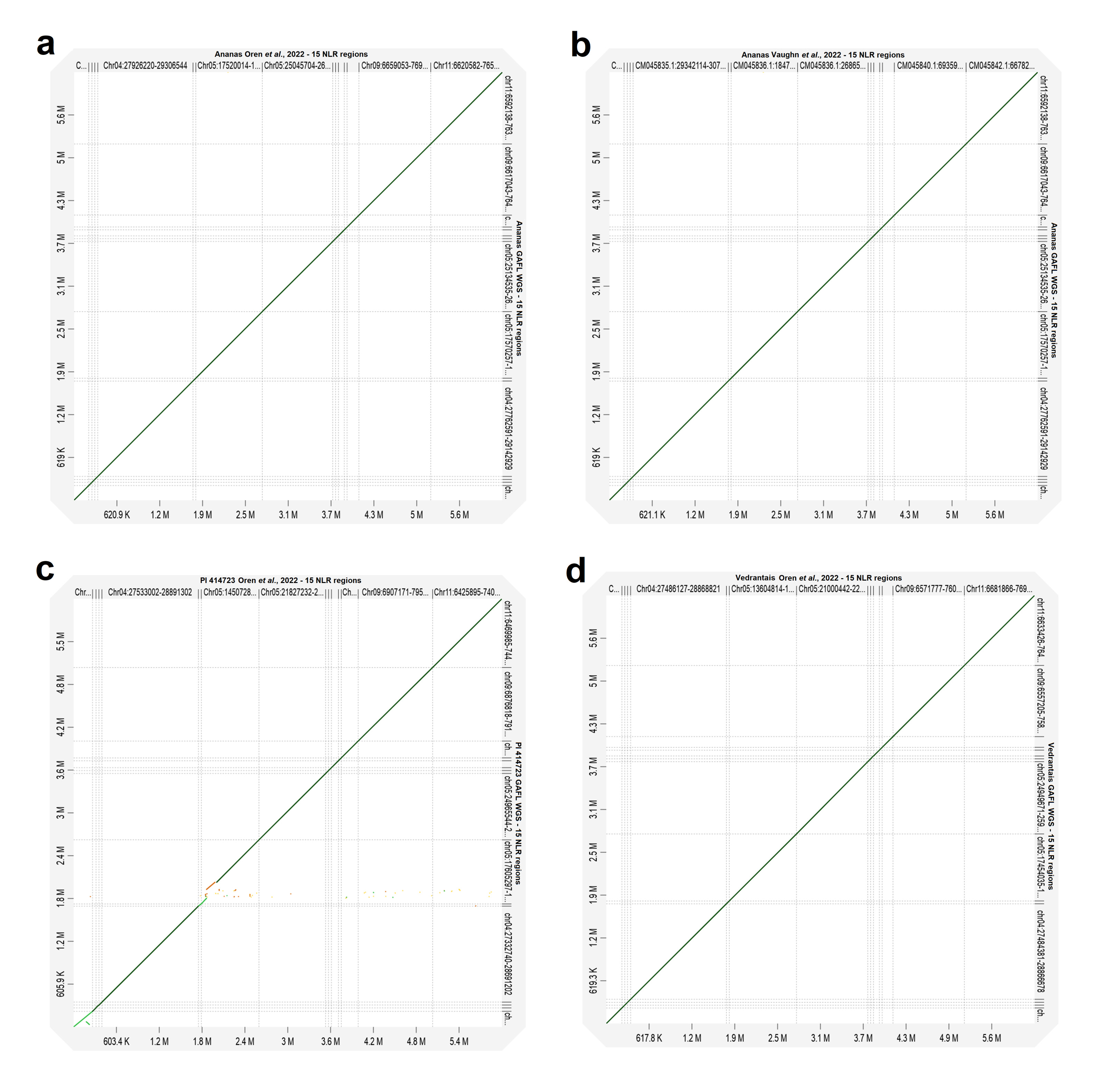


**Figure S9.** Region-to-region alignments between the 15 NLR clusters from the assemblies constructed here (y-axis) and those from the previously published genomes by Oren et al. (2022) and Vaughn et al. (2022) (x-axis). a) Ananas GAFL vs Ananas Oren et al. (2022). b) Ananas GAFL vs Ananas Vaughn et al. (2022). c) PI 414723 GAFL vs PI 414723 Oren et al., (2022). c) Vedrantais GAFL vs Vedrantais Oren et al. (2022). GAFL: “*Génétique et Amélioration des Fruits et Légumes*” research unit, Avignon, France.
